# Difference in the plasma level of miR‐628‐3p in atopic dermatitis patients with/without atopic keratoconjunctivitis

**DOI:** 10.1002/iid3.536

**Published:** 2021-09-21

**Authors:** Mayumi Ueta, Hiromi Nishigaki, Seitaro Komai, Chie Sotozono, Shigeru Kinoshita

**Affiliations:** ^1^ Department of Ophthalmology Kyoto Prefectural University of Medicine Kyoto Japan; ^2^ Department of Frontier Medical Science and Technology for Ophthalmology Kyoto Prefectural University of Medicine Kyoto Japan

**Keywords:** allergy processes, epigenetics processes, human, mucosa tissues

## Abstract

**Introduction:**

Some but not all patients with atopic dermatitis (AD) present with allergic conjunctival disease (ACD) including severe types such as atopic keratoconjunctivitis (AKC) with/without giant papillae. We hypothesized that different factors are involved in the severity of ACD and AD. Recently we reported that hsa‐miR‐628‐3p could affect the balance of innate immunity by suppressing pathogen‐associated molecular patterns such as toll‐like receptor 3 (TLR3), RIG‐I, and MDA‐5. We also reported that TLR3 positively regulates ocular surface‐ and skin inflammation such as contact dermatitis and AD. Here we compared the plasma level of miR‐628‐3p in AD patients with severe AKC with giant papillae and/or shield ulcers, with the level in healthy controls and AD patient without AKC or with very mild AKC.

**Methods:**

We used the plasma from 32 AD patients with severe AKC, from 40 healthy controls, and from 23 AD patient without AKC or with very mild AKC without giant papillae nor shield ulcers. Quantitative microRNA PCR assays were used to measure their plasma level of miR‐628‐3p.

**Results:**

We found that plasma miR‐628‐3p was upregulated in AD with severe AKC, but not in severe AD without severe AKC, nor in our healthy controls.

**Conclusion:**

Our new findings suggest that the plasma miR‐628‐3p level may represent a marker to predict the presence of severe AKC in AD patients.

**Abbreviations**
ACD: allergic conjunctival disease
AD: atopic dermatitis
AKC: atopic keratoconjunctivitis
MDA‐5: melanoma differentiation‐associated gene 5
RIG‐I: retinoic acid‐inducible gene‐I
SJS: Stevens‐Johnson syndrome
SOC: severe ocular complications
TARC: thymus and activation‐regulated chemokine
TEN: toxic epidermal necrolysis
TLR3: toll‐like receptor 3
VKC: vernal keratoconjunctivitis

## 1 INTRODUCTION

Some but not all patients with atopic dermatitis (AD) present with allergic conjunctival disease (ACD) including severe types such as atopic keratoconjunctivitis (AKC) with/without giant papillae. Elsewhere we suggested that the severity of AD is not necessarily proportional to the severity of ACD; not all patients with severe AD develop severe AKC with shield ulcers and/or giant papillae. Our earlier findings of the total immunoglobulin E (IgE‐), and the serum thymus and activation‐regulated chemokine (TARC) levels, and the history of shield ulcers in 5 patients with severe AD (serum TARC >1000 pg/ml) and 6 patients with severe AKC with giant papillae are shown in Table 1.

**Table 1:** Comparison between severe AD and severe AKC

Sex	Age	Total IgE (IU/ml)	TARC (pg/ml)	History of shield ulcers
Severe atopic dermatitis (severe AD)				
F	21	885	7380	No
M	16	15,208	3743	No
M	48	16,185	4889	No
M	45	19,582	1539	No
M	36	25,255	5039	No
average	33.2	15,423	4518	
Severe atopic keratoconjunctivitis (severe AKC)				
M	8	970	153	Yes
M	13	5078	739	Yes
M	47	2270	1539	Yes
M	15	758	690	Yes
M	15	1929	268	Yes
M	35	13,084	2264	Yes
average	22.2	4015	942	
*p* value (AD vs. AKC)		*p* < .05	*p* < .05	

Abbreviations: AD, atopic dermatitis; AKC, atopic keratoconjunctivitis; IgE, immunoglobulin E.

**Figure 1:**
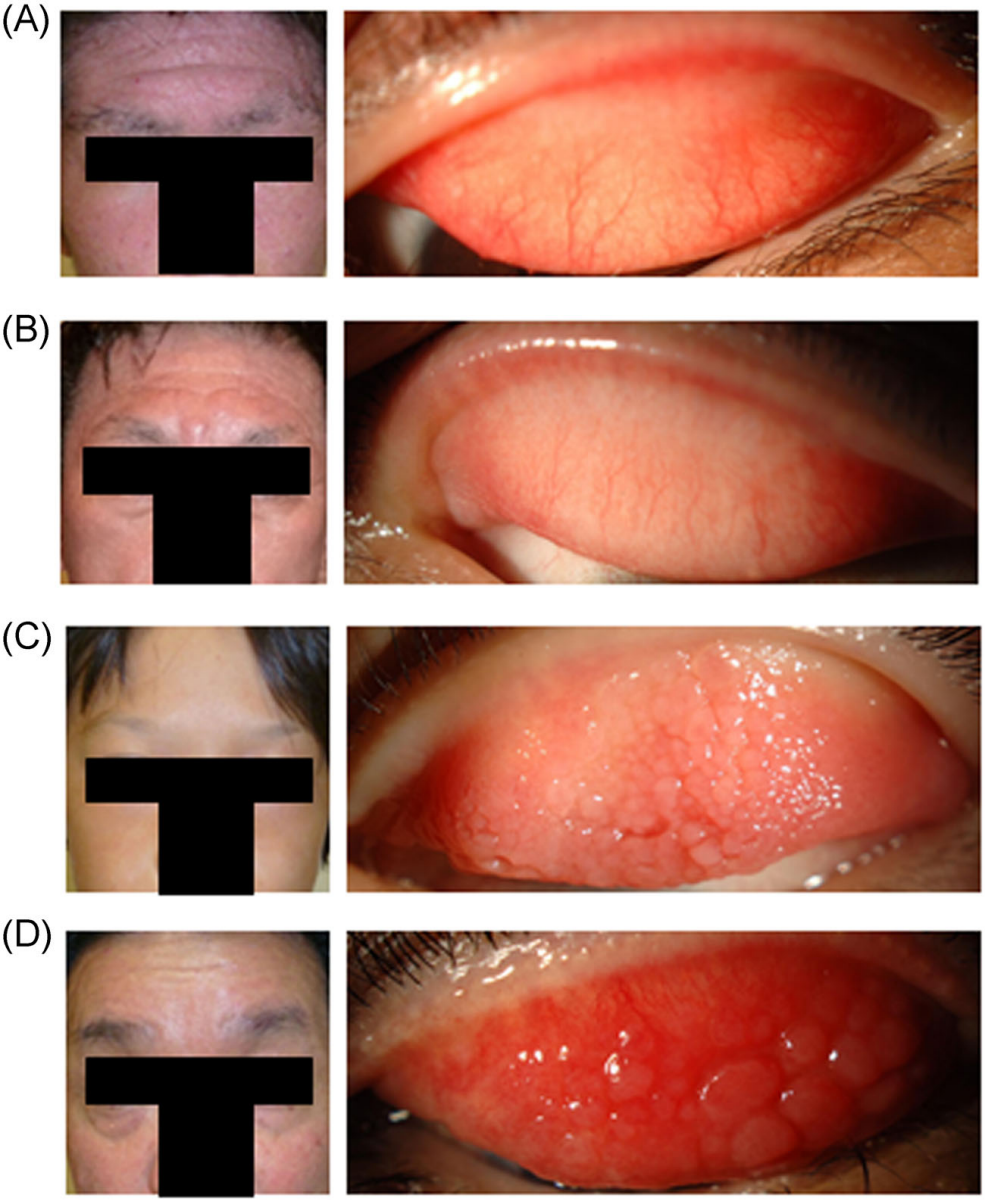
Photographs of the skin on the face and upper palpebral conjunctiva of representative patients. (A and B) Patients with severe AD treated by dermatologists with topical steroids and/or immunosuppressants. (C and D) Patients with severe AKC with giant papillae treated by ophthalmologists with eye drops of steroid eye drops and/or immunosuppressants. AD, atopic dermatitis; AKC, atopic keratoconjunctivitis

The five severe AD patients were treated with topical steroids and/or immunosuppressants by dermatologist in our hospital; the six severe AKC patients were treated with eye drops of steroid and/or immunosuppressant by ophthalmologists in our department. Figure 1 presents photographs of the skin on the face and the upper palpebral conjunctiva of representative patients. We found that the total IgE‐ and the serum TARC level was significantly higher in patients with severe AD than in AD patients with severe AKC with giant papillae. None of the five patients with severe AD and five of the six patients with severe AKC with giant papillae had history of shield ulcers. This suggests that it is not uncommon for patients to have severe AD but very mild ACD and that it is not uncommon for younger patients with severe AKC with giant papillae and/or shield ulcers to present with very mild AD treated with only moisturizers. Therefore, we posited the involvement of different factors in the severity of ACD and AD.

In our cornea clinic we encountered severe ACD with AKC or vernal keratoconjunctivitis (VKC) with shield ulcers and other severe ocular surface inflammatory diseases such as Stevens‐Johnson syndrome (SJS)/toxic epidermal necrolysis (TEN) with severe ocular complications (SOC). We have suggested that abnormal innate immunity might contribute to the pathogenesis of SJS/TEN with SOC.^1^ We also reported that hsa‐miR‐628‐3p was significantly upregulated in plasma from patients in the chronic stage of SJS/TEN with SOC and that miR‐628‐3p could regulate innate immunity by suppressing pathogen‐associated molecular patterns such as toll‐like receptor 3 (TLR3), RIG‐I, and MDA‐5.^2^ These findings support our hypothesis of abnormal innate immunity in patients with SJS/TEN with SOC.

Elsewhere we reported that TLR3 positively regulates ocular surface inflammation,^3^ skin inflammation such as contact dermatitis,^4^ and AD,^5^ and that the topical application of TLR3 inhibitors ameliorates chronic allergic skin inflammation in mice.^6^ These observations led us to investigate the plasma level of miR‐628‐3p, a potential TLR3 regulator, in AD patients with severe AKC with giant papillae and/or shield ulcers, in AD patients without AKC or with very mild AKC without giant papillae nor shield ulcers, and in healthy controls.

## 2 MATERIALS AND METHODS

This study was approved by the institutional review board of our institution; written informed consent was obtained from all patients; all received a detailed explanation of the purpose of the research and the experimental protocols. All procedures were conducted in accordance with the tenets of the Declaration of Helsinki. Included were 32 patients with severe AKC or VKC, who were treated for more than 6 months with the topical administration of the immunosuppressive drug tacrolimus and/or steroid eyedrops after the acquisition of blood samples. Also included were 23 AD patients without severe ACD who had not been treated or treated with only antiallergy eyedrops for eyes; 40 healthy individuals served as the control. Quantitative microRNA PCR assay was used to measure the plasma level of miR‐628‐3p.

## 3 RESULTS

**Figure 2:**
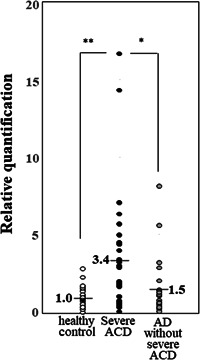
Plasma hsa‐miR‐628‐3p levels—quantitative miRNA PCR assays Comparison of the plasma hsa‐miR‐628‐3p level in patients with severe AKC versus patients with severe AD without severe AKC and our healthy controls. Quantification data were normalized to the expression of the internal control (miR‐39). The graph includes all data points to show the data distribution. Mean ± *SEM*: controls (*n* = 40): 1.00 ± 0.09; severe AKC with giant papillae (*n* = 32): 3.42 ± 0.65; AD without severe AKC (*n* = 23): 1.53 ± 0.39. *p < .05, **p < .005. AD, atopic dermatitis; AKC, atopic keratoconjunctivitis; miRNA, microRNA

Compared to the controls, the average plasma miR‐628‐3p level was 3.4 times higher in patients with severe AKC and 1.5 times higher in AD patients without severe AKC. There is a significant different between patients with severe AKC and controls (*p* < .005), but not between AD patients without severe AKC and controls (Figure 2). Moreover, there is also a significant different between patients with and without severe AKC (*p* < .05) (Figure 2).

## 4 DISCUSSION

Elsewhere we documented that plasma hsa‐miR‐628‐3p was significantly upregulated in the chronic stage of SJS/TEN with SOC and that miR‐628‐3p could regulate innate immunity by suppressing pathogen‐associated molecular patterns such as TLR3, RIG‐I, and MDA‐5.^2^ TLR3 is involved in the upregulation of ocular surface inflammation.^3^

We suspect that the hsa‐miR‐628‐3p increase in the plasma of SJS/TEN patients due to its systemic (plasma) upregulation compensates for its local (ocular surface) downregulation because it was downregulated in their conjunctival epithelium obtained at surgical ocular surface reconstruction. Although no conjunctival epithelium from patients with severe AKC was available, we also suspect that the increase in their plasma hsa‐miR‐628‐3p level may be related to its downregulation on their ocular surface.

It was reported that the level of tear eosinophil cationic protein, eotaxin2, and sIL‐6R was significantly higher in AKC patients than healthy controls.^7^ Earlier we reported that in the tears of AKC patients, as in patients with SJS, but not in healthy controls, interleukin (IL)−6, IL‐8, eotaxin, and macrophage inflammatory protein−1b were significantly upregulated and that the level of total IgE was significantly higher in AKC patients than in patients with SJS and in the healthy controls.^8^ We also found that stratum corneum TLR3 expression correlated with the severity of human AD lesions.^9^

To the best of our knowledge, ours is the first exploration of biomarkers that help to differentiate between AD with‐ and AD without severe AKC.

Allergic diseases include AD, ACD, asthma, and allergic rhinitis; some patients have AD and ACD, others only asthma, and yet others present with asthma and allergic rhinitis. As their combinations vary, different factors may be involved in the severity of each allergic disease. Because hsa‐miR‐628‐3p can regulate innate immunity, the allergic disease phenotype may play a role in the balance of innate immunity in patients with severe AD and patients with severe ACD. Our findings that plasma miR‐628‐3p was upregulated in AD with severe AKC, but not in severe AD without severe ACD, nor in our healthy controls, suggests that the plasma miR‐628‐3p level represents a marker to predict the presence of severe ACD in AD patients.

## CONFLICT OF INTERESTS

The authors declare that there are no conflict of interests.

## AUTHOR CONTRIBUTIONS

*Planning*: Mayumi Ueta; *experiments*: Mayumi Ueta and Hiromi Nishigaki; *analysis*: Mayumi Ueta, Hiromi Nishigaki, and Seitaro Komai; *writer of the manuscript*: Mayumi Ueta; *review of the manuscript*: Mayumi Ueta, Chie Sotozono, and Shigeru Kinoshita.

## Data Availability

The data that support the findings of this study are available from the corresponding author upon reasonable request.
